# Severe Fever with Thrombocytopenia Syndrome Virus, Shandong Province, China

**DOI:** 10.3201/eid1806.111345

**Published:** 2012-06

**Authors:** Li Zhao, Shenyong Zhai, Hongling Wen, Feng Cui, Yuanyuan Chi, Ling Wang, Fuzhong Xue, Qian Wang, Zhiyu Wang, Shoufeng Zhang, Yanyan Song, Jun Du, Xue-jie Yu

**Affiliations:** Shandong University School of Public Health, Jinan, China (L. Zhao, H. Wen, Y. Chi, F. Xue, Z. Wang, Y. Song, X-j Yu);; Zibo Municipal Center for Disease Control and Prevention, Zibo, China (S. Zhai, F. Cui, L. Wang, J. Du);; Yiyuan County Center for Disease Control and Prevention, Yiyuan, China (Q. Wang, S. Zhang);; University of Texas Medical Branch, Galveston, Texas, USA (X-j Yu)

**Keywords:** Fever, thrombocytopenia, SFTS virus, tick, goat, seroprevalence, Shandong, China, viruses, severe fever with thrombocytopenia syndrome virus, *Suggested citation for this article*: Zhao L, Zhai S, Wen H, Cui F, Chi Y, Wang L, et al. Severe fever with thrombocytopenia syndrome virus, Shandong Province, China. Emerg Infect Dis [serial on the internet]. 2012 June [*date cited*]. http://dx.doi.org/10.3201/eid1806.111345

## Abstract

Severe fever with thrombocytopenia syndrome, which results in severe illness and has a high case-fatality rate, is caused by a novel bunyavirus, severe fever with thrombocytopenia syndrome virus. We found that samples from 2/237 (0.8%) healthy persons and 111/134 (83%) goats in Yiyuan County, Shandong Province, China, were seropositive for this virus.

Severe fever with thrombocytopenia syndrome (SFTS) is a serious infectious disease with 12% case-fatality rate that has been documented in 6 provinces rural in northeast and central China. SFTS is caused by a novel bunyavirus, SFTS virus (SFTSV) (*1*). The major clinical signs and symptoms of SFTS are fever, thrombocytopenia, leukopenia, and elevated serum hepatic enzyme levels.

SFTSV is classified in the family *Bunyaviridae*, genus *Phlebovirus*, and is believed to be transmitted by ticks because the virus has been detected in *Haemaphysalis longicornis* ticks (*1*). However, the disease can also be transmitted from person to person through contact with infected patient’s blood or mucous (*2**,**3*). SFTSV seroprevalence in the human population is unknown, and the natural reservoir hosts of SFTSV have not been determined. We report results from a SFTSV serosurvey conducted on healthy persons and goats in Yiyuan County in Shandong Province, China, an area to which SFTS is endemic.

## The Study

Yiyuan County, located in eastern China (117°48′–118°31′E, 35°55′–36°23′N; Figure), has an area of 1,636 km^2^ and a population of 550,000 persons; 85% of the population is considered involved in agriculture. The county consists of low-lying hills with forests and grasslands and considered in a warm temperate zone, with a continental monsoon humid climate and 4 distinct seasons. The annual average temperature is 11.9°C, and the average rainfall is 720.8 mm. Farmers plant crops and fruit trees and raise goats as a major source of income in the area.

Most farm families have a herd of goats, and some of them have dogs. We found that goats and dogs in this county were heavily infested with ticks, with several hundred ticks found on each goat and dog. A recent serosurvey of domestic animals in Jiangsu Province found an SFTSV antibody positive rate of 57% in goats, 32% in cattle, 6% in dogs, 5% in pigs, and 1% in chickens (*4*). We selected goats for our seroprevalence survey because there was large population (n = 400,000) in Yiyuan County in 2011 (*5*) and because goats were heavily infested with ticks. Dogs were not surveyed because the small population of dogs made it difficult to obtain adequate sample numbers. Other domestic animals in the area (e.g., cattle, pigs, rabbits, and chickens) were not surveyed because they were usually raised in captivity.

The investigation of SFTSV seroprevalence was conducted during June 2011 in 10 rural villages in northwestern Yiyuan County with a total registered population of 7,406 (Figure). We recruited a convenience sample of 237 healthy volunteers from these villages and collected blood samples from all volunteers. The volunteers consisted of a small portion (3.2%) of the farmer population in the villages. A standardized questionnaire was used to obtain information on age, sex, history of illness, tick exposure, and occupation of each participant. All study participants were goat farmers who were also involved in agriculture and were longtime village residents. The research protocol was approved by the human bioethics committee of Shandong University, and all participants provided written informed consent.

**Figure:**
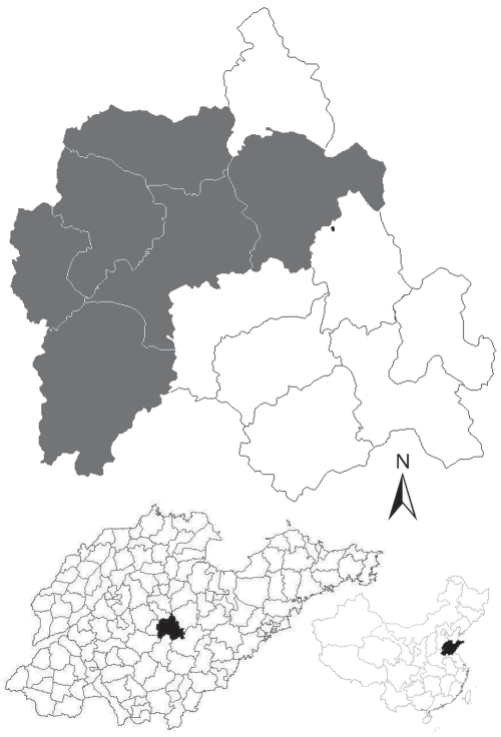
Location of the villages (gray shading) in Yiyuan County, Shandong Province, China, where human and goat serum samples were collected in study of severe fever with thrombocytopenia syndrome seroprevalence. Maps at bottom show location of Yiyuan County in Shandong Province (left) and Shandong Province in China (right).

Participant age ranged from 20 to 80 years (median 54 years); 150 (63%) were female. No volunteer was <20 or >81 years of age. The age and sex distribution of the study population may have resulted from the migration of men and young people from rural areas to cities or lower participation among older and younger person.

Serum samples were tested for total antibodies (IgG and IgM) to SFTSV by using a double-antigen sandwich ELISA kit, provided by Jiangsu Province Center for Disease Control and Prevention (*5*). The ELISA kit used recombinant nucleoprotein (NP) of SFTSV as an antigen, which was coated onto a plate. In the preliminary experiment, undiluted serum (50 µL) was added to a well of the plate, and the plate was incubated for 30 min at 37°C to enable SFTSV antibodies to bind to NP of SFTSV antigen. After washing, the bound SFTSV antibodies were reacted with horseradish peroxidase–labeled recombinant SFTSV NP and detected by substrates for horseradish peroxidase.

Absorbance of the plate was read at 450 nm. A sample was considered positive to SFTSV when the absorbance of the serum sample was >2.1× the absorbance of the negative control (provided by the manufacturer), which was 3 SD above the mean optical density at 450 nm for the persons sampled. The ELISA had similar specificity and sensitivity to the microneutralization assay and exhibited no cross-reactivity with hantavirus or dengue virus antibodies (*4*)

ELISA detected SFTSV antibodies in 3 healthy persons when undiluted serum samples were used. The positive samples were diluted to determine the antibody titers, and 2 serum samples were positive after dilution (Table 1). This finding could indicate a false-positive result for the serum that became negative upon dilution. Therefore, only the 2 persons whose samples remained positive after dilution were considered seropositive for SFTSV. Thus, the seroprevalence of SFTSV in the investigated population was 0.8% (2/237) (Table 1). Both of these persons were female, and neither reported SFTS symptoms in the past, being hospitalized for any clinically similar disease, or contact with a person who had fever and thrombocytopenia syndrome as defined (*1*).

**Table 1:** Age distribution of anti–severe fever with thrombocytopenia syndrome virus IgG/IgM detected in healthy human volunteers from rural area of Yiyuan County, Shandong Province, China

Age group, y	No. persons tested	No. seropositive by ELISA (titer)
0–20	0	0
21–30	4	1 (128)
31–40	19	0
41–50	77	0
51–60	76	1 (512)
61–70	34	0
71–80	27	0
>81	0	0
Total	237	2

We also collected blood samples from 134 goats from 16 herds in 8 villages in the county during June and August 2011. Most famers in the area have goats, but few volunteered to donate their goats’ blood for our study. Therefore, we selected all 16 herds whose owners allowed us to sample their goats and randomly sampled 2–20 goats from each herd (the number of goats sampled was determined by the owner). The goat serum samples were tested for total antibodies to SFTSV by using the double-antigen sandwich ELISA kit as described human samples. Of the 134 goats sampled, 111 (83%) had antibodies to SFTSV according to the established cutoff of >2.1× the absorbance of the negative contro. We further diluted the goat serum in 2-fold increments, from 1:8 to 1: 512, and the titers were all >32 (Table 2).

**Table 2:** Anti–severe fever with thrombocytopenia syndrome virus IgG/IgM detected in serum samples from healthy goats from rural area of Yiyuan County, Shandong Province, China

ELISA titer	No. positive serum samples
32	9
64	24
128	25
256	10
>512	43
Total	111

## Conclusions

We found a high seroprevalence of SFTSV among goats in Yiyuan County but a low seroprevalence among humans. The seropositive persons were from 2 different villages and were not linked to each other; only 1 was involved in herding goats and neither in milking or butchering. Thus, the exposure route was not clear. Neither of the seropositive persons reported clinical illness; however, this finding is limited by the possibility of recall bias.

A previous study of 200 serum samples collected from SFTSV-endemic areas showed no subclinical infection with SFTSV (*1*). The discrepancy between that study and our study may be a result of the low overall seroprevalence of SFTSV. We conclude that subclinical SFTSV infections or a relatively mild form of SFTS illness may occur in humans; however, more research is needed.
